# Integrated Network Pharmacology and Mice Model to Investigate Qing Zao Fang for Treating Sjögren's Syndrome

**DOI:** 10.1155/2022/3690016

**Published:** 2022-01-10

**Authors:** Ping Zeng, Wei Liu, Shumin Zhang, Shaopeng Du, Yihua Fan, Longmei Zhao, Aihua Wang

**Affiliations:** ^1^First Teaching Hospital of Tianjin University of Traditional Chinese Medicine, Tianjin 300193, China; ^2^National Clinical Research Center for Chinese Medicine Acupuncture and Moxibustion, Tianjin 300381, China; ^3^Tianjin University of Traditional Chinese Medicine, Tianjin 301617, China; ^4^Guizhou University of Traditional Chinese Medicine, Guiyang 550025, China

## Abstract

Sjögren's syndrome (SS) is an autoimmune disease, and its conventional treatment has exhibited limited therapeutic efficacy. Qing Zao Fang (QZF), a traditional Chinese medicine formula, is used in the treatment of Sjögren's syndrome, but its chemical composition is complex, and its pharmacological mechanism is not clear. Therefore, this study aims to explore the potential mechanism of QZF in the treatment of Sjögren's syndrome based on network pharmacology and SS mouse model. The main active components and predicted targets of QZF were analyzed by network pharmacology. The SS mouse model was constructed and divided into 6 groups: control, SS, SS + hydroxychloroquine (HCQ)-treated, SS + low-dose QZF-treated, SS + medium-dose QZF-treated, and SS + high-dose QZF-treated group. Immunohistochemical, ELISA, and qRT-PCR assays were performed to detect the expressions of targets associated with SS. TUNEL staining was used to detect apoptosis. Cumulatively, 230 active compounds and 1883 targets of QZF were identified. There were 227 common targets for QZF and SS. The effective active ingredients were stigmasterol, neocryptotanshinone II, neotanshinone C, miltionone I, and beta-pinene. It mainly acts on biological processes such as inflammatory response, chemokine metabolic process, and immune response as well as pathways such as FoxO signaling pathway, *Yersinia* infection, HIF-1 signaling pathway, and TNF signaling pathway. In SS mice, levels of AKT1, HIF-1*α*, TNF-*α*, IL-6, and IL-17A were increased, while decreased after QZF treatment. In contrast, IL-10 levels were decreased in SS mice and increased in QZF-treated mice. In addition, QZF reduced apoptosis in the submandibular gland tissue compared to SS mice. It can be concluded that the QZF in treatment of SS is the result of the combined action of multiple components, multiple targets, and multiple pathways. This study improves the understanding of the link between QZF and SS on molecular mechanisms.

## 1. Introduction

Sjögren's syndrome (SS) is well-recognized as a systemic autoimmune disease, and it is characterized by manifestations of sicca (dryness) of the oral, ocular mucosa, and genital taking diverse exocrine glands as targets [1]. This disorder is categorized into primary SS existing as an independent entity and secondary SS with a coexisting entity [2]. Symptoms of secondary SS are aggravated accompanying with additional autoimmune diseases including systemic lupus erythematosus, systemic sclerosis, or rheumatoid arthritis [3]. The spread of this disorder can cause several organic extraglandular manifestations of the skin, thyroid, liver, lungs, kidneys, gastrointestinal tracts, and nervous systems as well [3, 4]. So far, studies have suggested that the incidence of SS is related to genetics, viral infection, related cytokines, neurohormone disorders, and environmental factors, yet it is still insufficiently understood, diagnosed, and treated [5].

As a chronic autoimmune disease, the pathogenesis of SS remains insufficiently identified. Numerous experiments have shown that B cells collaborate with T cells to activate in response to autoantigens in this disease, resulting in lymphocyte infiltration and immune complex deposition [6]. Results of function analysis using murine SS models have elucidated that Th1, Th2, and Th17 cells are of great significance in the occurrence and development of SS, which play roles in regulating the differentiation, amplification, and functions of self-reactive T and B cells [7–9]. Furthermore, inflammatory cytokines balance the epithelial homeostasis and modulates the functions of B and T cells [3]. Ectopic expressions of CXC chemokines (CXCL9, CXCL10, CXCL11, and CXCL13), interleukins (IL-1/2/4/6/10/12/17/22/23), IFN-*γ,* and TNF-*α* occur in the minor salivary glands of SS patients as compared to healthy individuals [10, 11].

Current therapeutic options for SS mainly relies on improving mouth and eye dryness and suppressing immune responses, such as artificial tears and saliva substitutes, nonsteroidal anti-inflammatory drugs, glucocorticoids, hydroxychloroquine, synthetic immunosuppressive agents, and T or B cell-targeting biological therapies [12]. Despite alleviation of those sicca symptoms, clinical treatment of SS remains a challenge: a specific therapeutic target as the most important goal is in urgent need beyond symptom relief [12]. In light of the principle of the traditional Chinese medicine (TCM), SS is classified into the “dryness arthralgia” category, and the symptoms has been demonstrated to be alleviated, guiding under the TCM pathogenesis of “deficiency, stasis, and toxicity” [13]. Some herbal formulae or single herb have been proved to play roles in reducing IFN-*γ* and IL-4 contents in the peripheral blood and increasing the secretion of saliva of SS patients [14, 15]. Xuan-Shen (S*crophularia ningpoensis*), Mai-Men-Dong (*Ophiopogon japonicus*), and Sheng-Di-Huang (*Rehmannia glutinosa*) were the widely introduced single herbs for treating SS [16]. Since the concept of network pharmacology was first proposed by Hopkins [17] as part of bioinformatics, it has been extensively applied in relevant studies of complex TCM compounds. The reason lies in the fact that network pharmacology combines system network analysis and pharmacology and simultaneously contains the connotation of holistic theory of TCM with multicomponents, multitargets, and multipathways [18].

According to the pathogenesis characteristics of SS, we composed QZF based on the theory of “deficiency, stasis, and toxicity.” This study intends to systematically summarize the potential effective components and action pathways of QZF in SS treatment using analysis of network pharmacology and mouse model experiments in vivo. It is expected to provide an insight into the clinical applications using pharmacological analysis for the treatment of SS.

## 2. Materials and Methods

### 2.1. Network Pharmacology

#### 2.1.1. Active Components and Putative Targets of QZF

The bioinformatic platforms BATMAN-TCM (http://bionet.ncpsb.org/batman-tcm/) and TCMSP (http://lsp.nwu.edu.cn/tcmsp.php) were utilized to obtain the active components and putative targets of QZF [19, 20]. QZF is made of 10 herbs: Mai Dong (*Ophiopogon japonicus*) 10 g, Shi Hu (*Herba Dendrobii*) 10 g, Yu Zhu (*Polygonatum odoratum*) 10 g, Sheng Di Huang (*Rehmannia glutinosa*) 10 g, and Gan Cao (*Glycyrrhiza uralensis*) 6 g, and so on. The scores of predicted candidate targets (including known targets) which were not smaller than the score cutoff 20 of the ingredients were presented. ID information of the active components were obtained from the PubChem (https://pubchem.ncbi.nlm.nih.gov/).

#### 2.1.2. Known SS-Related Targets

We input “Sjögren's syndrome” as the keyword in the GeneCards database (https://www.genecards.org/) to sort out SS-related targets [21]. The predicted targets of QZF and targets of SS were subsequently uploaded to the SangerBox data analysis platform (http://sangerbox.com/) to obtain their common targets.

#### 2.1.3. Network Construction

To further investigate the potential mechanisms of QZF on SS, we constructed protein-protein interaction (PPI) network diagrams through the Search Tool for the Retrieval of Interacting Genes (STRING) database (https://string-db.org/) [22]. The CentiScaPe plug-in tool of software Cytoscape 3.6.1 was applied for the calculation of degree centrality (DC) of each node, and the top 20 DC values of the core targets were chosen for further analysis. The values of “degree” represented the number of edges connected to a node and the importance representative of each node [23]. The diagram construction of the QZF compound-target network was completed using the software Cytoscape 3.6.1 [24].

#### 2.1.4. Gene Ontology (GO) and Kyoto Encyclopedia of Genes and Genomes (KEGG) Analysis

Following import of the 20 core targets into Cytoscape, analysis of GO biological process and KEGG pathway enrichment were subsequently conducted by using the ClueGO plugin at an adjusted *p* values of <0.05 [25]. Target protein analysis was performed using the GO analysis with the entries of the top 20 functional categories from biological process (BP), cellular component (CC), molecular function (MF), and immune system process (IS). The GO and KEGG analysis results were uploaded to SangerBox for visualization.

### 2.2. Induction of SS in Mice

The animal experiments gained approval of the Guiyang Second Affiliated Hospital of Traditional Chinese Medicine. The spontaneous SS model in NOD mice were used for experimental study which is naturally produced under natural conditions without artificial induction or due to genetic mutation, and often represents a mouse model that has been retained through genetic breeding [26]. A total of 40 SPF female NOD mice aged 8 weeks and 8 eight-weeks-old SPF female BALB/c mice were sourced from Chongqing Tengxin Biotechnology Co., Ltd (Chongqing, China). The animals were maintained in specific pathogen-free animal houses. The SS mouse model was described by Lin et al. [9]. The blank control group used the BALB/c mice. The NOD mice were randomly divided into 5 groups (8 each): SS, SS + HCQ-treated, SS + low-dose QZF-treated, SS + medium-dose QZF-treated, and SS + high-dose QZF-treated group.

### 2.3. Administration Method

QZF is the experience prescription and provided by the pharmacy of the Guiyang Second Affiliated Hospital of Traditional Chinese Medicine. It was formulated based on the basic theories of traditional Chinese medicine and clinical experience to treat Sjögren's syndrome. The toxicity and adverse effects induced by QZF were detected: there is a certain possibility of causing central obesity, elevated fasting blood sugar, insomnia, and elevated blood pressure [27]. Mice in each group were fed adaptively for 2 weeks and then given intragastric treatment. QZF in treating primary Sjögren's syndrome was studied in clinic, the dosage of QZF was determined in the previous literature, and according to the body weight of human and animal, low-dose QZF water decoction (14.5 g kg^−1^/d), medium-dose (29 g kg^−1^/d), and high-dose (58 g kg^−1^/d) were given orally, once a day, respectively [28, 29]. The positive control group was given hydroxyl chloroquine (40 g kg^−1^/d) water solution for consecutive 6 weeks, while an equal amount of normal saline (0.2 mL/d) was given to both the blank and SS model control groups by gavage. After four weeks of continuous administration, the mice were anesthetized and sacrificed using pentobarbital sodium at 50 mg/kg as per body weight. Blood samples from mice eyeballs and submandibular gland tissue were collected for subsequent experiments.

### 2.4. Quantitative Real-Time Polymerase Chain Reaction (qRT-PCR)

The mouse submandibular gland was prepared for total RNA extraction using the TRNzol-A reagent (Tiangen, Beijing, China). The Primer Script RT Reagent Kit was employed for reverse transcription synthesis of cDNA (Beijing Kangrun Chengye Biotechnology Co., Ltd, Beijing, China) as per the manufacturer's instructions of use. qRT-PCR was carried out on an ABI 7500 instrument (Thermo Fisher Scientific, Inc.) using 2 × Taq PCRStar Mix (Beijing Kangrun Chengye Biotechnology Co., Ltd, Beijing, China). The reaction system was as follows: 95°C for 30 sec, followed by 40 cycles of 95°C for 5 sec, 55°C for 30 sec, and 72°C for 30 sec. *β*-actin was then utilized as an internal control and repeated 3 times for each sample.

### 2.5. Enzyme-Linked Immuno Sorbent Assay (ELISA)

Next, we identified the expression of SS-related protein in serum using the ELISA kit based on the manufacturer's instructions. The ELISA kits of IL-1, IL-2, IL-6, IL-10, IL-17A, and IFN-*γ* were purchased from MultiSciences (LiankeBio) Biotech Co., Ltd. (Hangzhou, China). Briefly, 100 *μ*l sample was added to wells and incubated for 2 h at room temperature (RT). Each well was aspirated and washed five times before adding antibody (100 *μ*l), then incubated for 1 h at RT, and aspirated and wash five times again. Working reagent (100 *μ*l) was added, incubated for 30 min at RT, and aspirated and washed. One-step substrate reagent (100 *μ*l), 3,3′,5,5′tetramethylbenzidine (TMB) was added and incubated for 30 min at RT. Stop solution (50 *μ*l) was added and the absorbance was read at 450 nm within 30 min.

### 2.6. Immunohistochemistry

The salivary gland tissue fixation was conducted using 4% paraformaldehyde (Solarbio, Shanghai, China) and followed by paraffin embedment to make 5 *μ*m sections. Following deparaffinization, the sections were incubated with 3% H_2_O_2_ (Sinopharm, China) for 10 min and 0.1% trypsin (Beyotime, China) for 20 min, followed by primary antibodies incubated at 4°C overnight. PBS was used for the treatment of the negative control instead of primary antibodies. Then, the sections were incubated at 37°C for 30 min with HRP-conjugated secondary antibodies. Finally, the bound antibodies were stained with diaminobenzidine (DAB, Zhongshan, Beijing, China) and followed by hematoxylin (Jiancheng, Nanjing, China) counterstaining of the sections. An inverted microscope (Olympus, Japan) was employed for image acquisition. The primary antibodies anti-IL-6, anti-IL-10, and anti-IL-17 were purchased from Abcam.

### 2.7. Western Blot

The submandibular gland tissue was initially extracted and cell lysis buffer (Beyotime Biotechnology, Jiangsu, China) was used to add 1 mM phenylmethanesulfonyl fluoride (Beyotime Biotechnology, Jiangsu, China) to extract protein lysates. The cell membrane and cytoplasm was obtained by cell disruption. The protein samples of different molecular weights were separated by 10% SDS/PAGE and transferred into polyvinylidene difluoride membranes (Millipore, Billerica, MA). Then, the samples were washed with TBST and blocked with phosphate-buffered saline (PBS) containing 5% blotting-grade blocker for 1 h. Followed by blocking, three times washing, each for 5 min, was performed with tris-buffered saline with Tween 20 (TBST). The appropriate primary antibody (1 : 1000) incubation was performed on the transferred membrane at 4°C overnight and then followed by 1 h IgG (1 : 2000, ABclonal) incubation at room temperature. Proteins bands were visualized with ultrasensitive chemiluminescence kits (10300, New Cell & Molecular Biotech Co, Ltd, Suzhou, China). The catalogue number and brand of used primary antibodies were listed as follows: anti-Bcl-2 (#2876, Cell Signaling Technology), anti-Bax (#2772, Cell Signaling Technology), and *β*-actin (ab8227, Abcam). Also, anti-IL-6, anti-IL-10, and anti-IL-17 were purchased from Abcam.

### 2.8. TUNEL Assay

Following three cycles of washing with PBS, the sections of salivary gland tissue cells were stained using TUNEL kits [cat. no. 11684817910; Roche Diagnostics (Shanghai) Co., Ltd.] as per the manufacturer's instructions of use. Observation of apoptotic cells was performed under an inverted fluorescence microscope (cat. no. CKX53; Olympus Corporation) at magnification ×400 folds. Samples in four fields were randomly calculated, and brown color represented the apoptotic cells, and blue, the nucleus pulposus.

### 2.9. Statistical Analysis

Data obtained from the previously described procedures were presented as mean ± standard deviation. All experiments were independently duplicated three times. Statistical analyses of the data were performed using the SPSS 23.0 software (IBM Corp.). One-way analysis of variance (ANOVA) was employed for comparison among multiple groups followed by Tukey's post hoc test. *p* < 0.05 denoted that the difference was statistically significant.

## 3. Results

### 3.1. Compounds and Potential Targets of QZF

**Figure 1:**
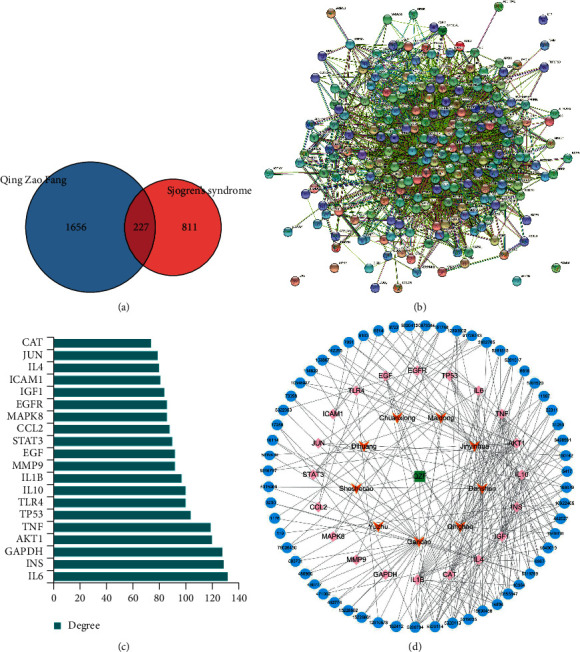
The active components and potential targets of Qing Zao Fang (QZF) in the treatment of Sjogren's syndrome (SS). (a) Venn diagram of common targets of QZF and SS. (b) Protein-protein interaction (PPI) network analysis of 227 common targets. (c) The top 20 targets with the largest DC value in PPI networks. (d) The compound-target network of QZF in the treatment of SS. Blue nodes represent the 59 active components of QZF, pink nodes represent the 20 core targets, and orange nodes represent the corresponding 9 kinds of herbs.

Screening of active components and targets of QZF were conducted using the platforms of BATMAN-TCM and TCMSP, and 230 active components and 1883 predictive targets were obtained. Through GeneCards retrieval, 1038 SS target genes were collected, and the corresponding targets of each component of QZF were intersected with targets of SS. There were 227 common targets between the QZF and SS (Figure 1(a)). PPI network graphs of 227 common targets were obtained using the String website (Figure 1(b)). The degree centrality (DC) value of PPI was calculated by the software Cytoscape 3.6.1. to screen out the 20 targets with the largest DC value, among which IL-6, INS, GAPDH, AKT1, and TNF may be the most core targets of QZF in the treatment of SS because of the highest DC value (Figure 1(c)). The compound-target network of QZF was established (Figure 1(d)). Only 9 herbs and 59 active components corresponded to the 20 common targets. Among the active compounds 5280794 (stigmasterol), 15690458 (neocryptotanshinone II), 5320114 (neotanshinone C), 5319835 (miltionone I), and 14896 (beta-pinene) had the highest DC values. Taken together, the compounds presenting the highest relative positioning DC values were considered as the potential key active compounds.

### 3.2. GO and KEGG Pathway Enrichment Analysis

**Figure 2:**
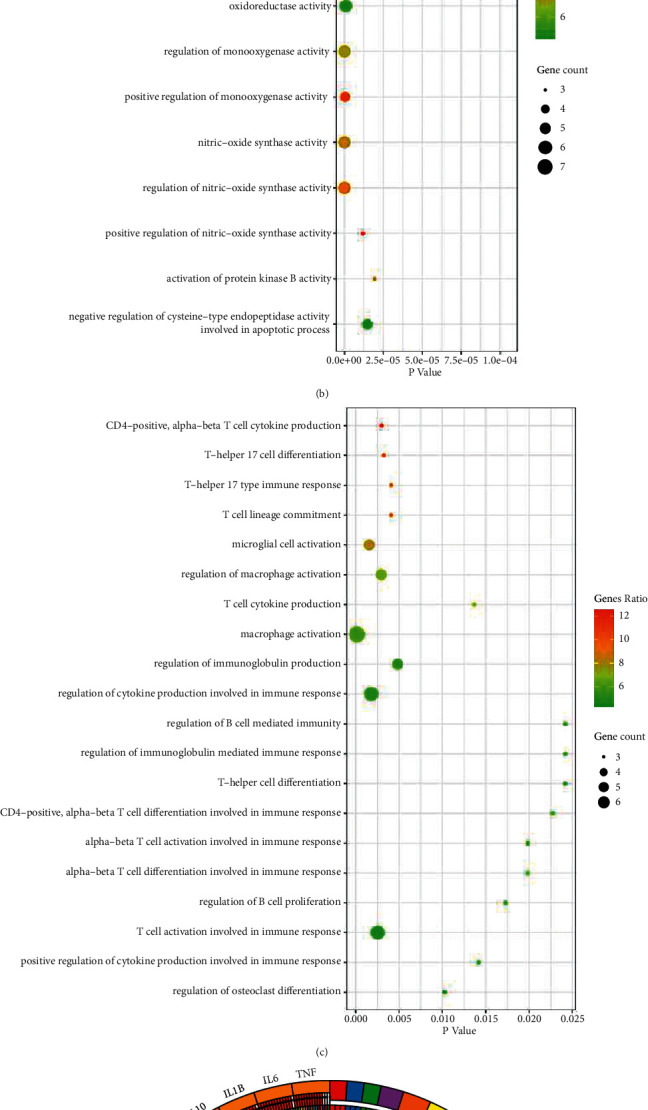
GO and KEGG pathway enrichment analysis of candidate targets of QZF against SS. (a) Gene ontology terms of the targets in biological process, (b) Immune system process, and (c) Molecular function. (d) KEGG pathway enrichment of the candidate targets. The number of genes enriched in this GO term accounts for the percentage of 20 core targets, and the gene ratio magnitude was represented by green-red color. Gene count was represented by circle size which means number of genes enriched in this GO term.

To verify whether QZF in treating SS produced any biological responses, we performed GO analysis on the 20 SS-related potential therapeutic target genes on the processes of BP, CC, IS, and MF. After filtering by adjusted *p* value < 0.05, 20 GO terms were displayed. In BP enrichment analysis, most terms are related with inflammatory response, gene silencing, and chemokine metabolic process (Figure 2(a)). CC was not shown in this article because only one term was related. In IS enrichment analysis, CD4-positive T cell cytokine production, T-helper 17 cell differentiation, and T-helper 17 type immune response presented the highest gene ratio value (Figure 2(b)). In MF enrichment analysis, terms were related with positive regulation of NF-*κ*B transcription factor activity, nitric oxide synthase activity, and oxidoreductase activity (Figure 2(c)). These targets were enriched in 56 pathways, and the adjusted *p* value was less than 0.05. The KEGG enrichment analysis of the top 20 entries is shown in Figure 2(d). The top four pathways were the FOXO signaling pathway (AKT1/CAT/EGF/EGFR/IGF1/IL-10/IL-6/INS/MAPK8/STAT3), *Yersinia* infection (AKT1/CCL2/IL-10/IL-1B/IL-6/JUN/MAPK8/TLR4/TNF), TNF signaling pathway (AKT1/CCL2/ICAM1/IL-1B/IL-6/JUN/MAPK8/MMP9/TNF), and HIF-1 signaling pathway (AKT1/EGF/EGFR/GAPDH/IGF1/IL-6/INS/STAT3/TLR4). TNF, IL-6, IL-1B, and IL-10 were the top four targets.

### 3.3. Effect of QZF on SS-Related Gene

**Figure 3:**
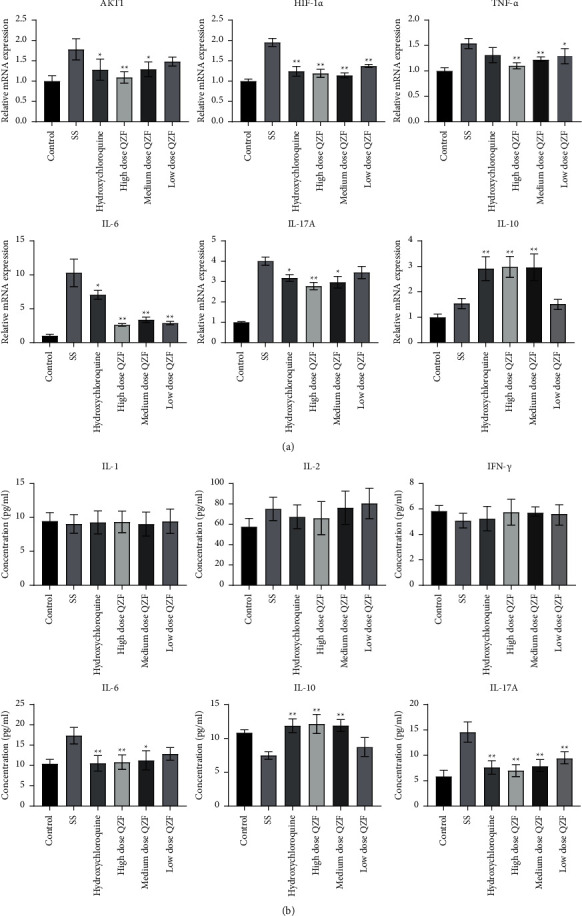
Effect of QZF on SS-related candidate targets at mRNA and protein levels. (a) SS-related candidate genes were measured by qRT-PCR. (b) SS-related candidate proteins were measured by ELISA assay. Comparisons among multiple groups were made with ANOVA followed by Tukey's post hoc test. ^*∗*^*p* < 0.05; ^*∗∗*^*p* < 0.01; compared to the SS mice model group.

Most of the cytokines that target SS contain the TNF family, IFN family, IL-1, IL-2, IL-6, IL-10, and IL-17 [3]. Hydroxychloroquine is the main medicine for the treatment of SS patients. To detect whether QZF have protective effects against SS, the SS mice model was established. In combination with the published literature and pharmacological analysis, the SS-related gene was measured by qRT-PCR. Results showed that AKT1, HIF-1*α*, IL-6, IL-17A, and TNF-*α* levels was markedly elevated in the serum of SS mice compared to those of the control. Their levels were significantly downregulated in the QZF- or HCQ-treated group compared with those of the SS model group, while IL-10 levels significantly decreased in serum from SS mice and upregulated in the QZF- or HCQ-treated SS mice. Besides, this effect did not show significant dose-dependence (Figure 3(a)).

**Figure 4:**
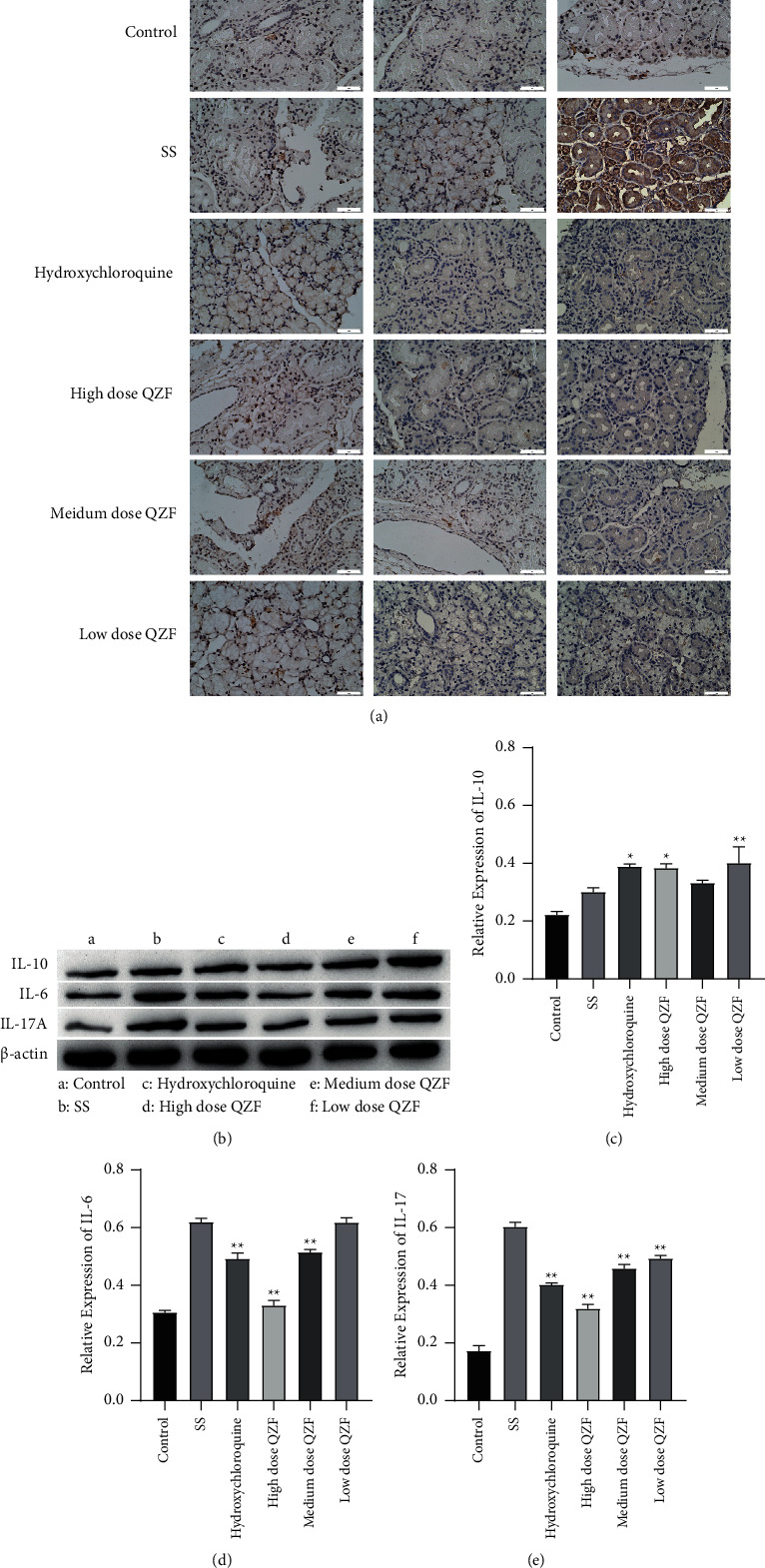
Effect of QZF on IL-6, IL-10, and IL-17A levels in submandibular gland tissue. (a) Representative immunohistochemical staining micrographs for IL-6, IL-10, and IL-17A; Bar = 50 *μ*m. (b) Western blot detection of the protein expressions of IL-6, IL-10, and IL-17A, and (c)–(e) shown as diagram with statistical analysis. Comparisons among multiple groups were made with ANOVA followed by Tukey's post hoc test. ^*∗*^*p* < 0.05, ^*∗∗*^*p* < 0.01; compared to the SS mice model group.

SS-related proteins were determined using ELISA and immunohistochemistry. The ELISA results indicated that levels of IL-17A and IL-6 were downregulated in the QZF-treated group in comparison to those of the SS model group whereas the IL-10 level was elevated in the QZF-treated group. IL-1, IL-2, and INF-*γ* showed no significant difference between the SS model group and the QZF-treated group. This may be because that the SS is a systemic autoimmune disease which mainly involves exocrine invasion, more significant inflammation may be observed in the exocrine glands (Figure 3(b)). At the same time, the specific mechanism of IL-1, IL-2, and INF-*γ* in SS has been studied in some research works [30], and the current clinical treatment of SS is more focused on IL-17 [31]; therefore, we concentrate more on IL-17 and research on HIF-1*α*. In the light of the above results, we detected the levels of IL-6, IL-10, and IL-17A in the submandibular gland tissue of mice by immunohistochemistry, which was consistent with the ELISA assay (Figure 4(a)). Furthermore, the IL-6, IL-10, and IL-17A protein expression in the submandibular gland tissue was detected by WB as well. The results showed the QZF can significantly decrease the IL-6 and IL-17A expression and promote the expression of IL-10 compared with that of SS (Figures 4(b)–4(e)).

### 3.4. Effect of QZF on Apoptosis of Submandibular Gland Tissue

**Figure 5:**
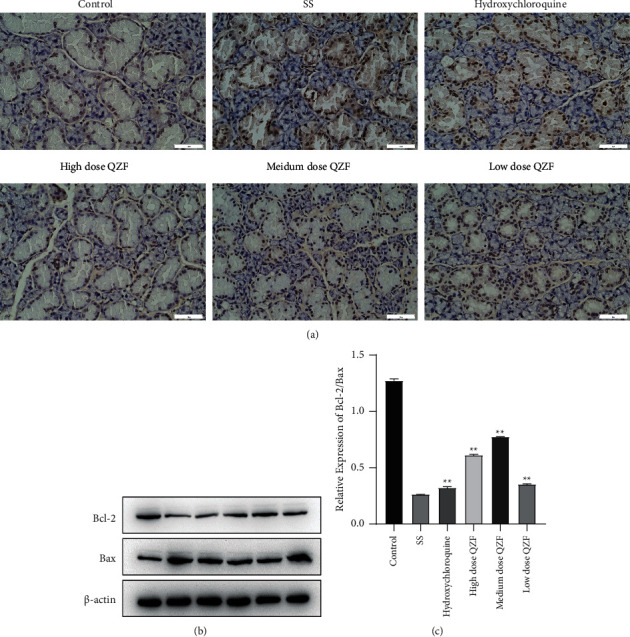
Effect of QZF on apoptosis in submandibular gland tissue. (a) QZF reduced apoptosis of the submandibular gland tissue in SS mice as measured by TUNEL assay; Bar = 50 *μ*m. (b) Western blot detection of Bcl-2 and Bax. (c) The Bcl-2/Bax ratio diagram with statistical analysis. Comparisons among multiple groups were made with ANOVA followed by Tukey's post hoc test. ^*∗∗*^*p* < 0.01; compared to SS mice model group.

We further examined whether QZF could affect the apoptosis of submandibular gland tissue. Apoptosis was measured by using the TUNEL assay and Bcl-2/Bax ratio in the submandibular gland tissue of mice that were treated or not with QZF. Bax gene is a proapoptotic member of the Bcl-2 family, and dimerization among members is an important form of functional regulation. Bax and Bcl-2 heterodimer can inhibit Bcl-2 and play a role in promoting cell apoptosis. As shown in Figure 5(a), the TUNEL-positive cells were observed in the SS group, and high-dose QZF can decrease the positive cells. The Bcl-2/Bax ratio was significantly increased in QZF-treated mice compared with SS mice, and the apoptosis was inhibited (Figures 5(b) and 5(c)). The results suggested that apoptosis of the submandibular gland tissue of SS mice was substantially increased, while it was greatly decreased after QZF or HCQ treatment (Figure 5). This implied that QZF has a positive effect on SS mice.

## 4. Discussion

TCM has named the SS as either “dry-Bi” or “dryness impediment.” Our preliminary studies have demonstrated that most SS patients present features of Qi and Yin deficiency including manifestation of blood stasis [14]. Some clinical trials have confirmed that SS treatment using TCM combined with Western medicine exerts a favorable effect on the disease, allowing to nourish Yin, supplement Qi, and activate blood circulation [14]. In accordance with this theory, we formulated QZF (Mai Dong, Shi Hu, Yu Zhu, Sheng Di Huang, Gan Cao, and so on) which could nourish Yin and moisten dryness. The top 3 single herbs for SS treatment in Taiwan are known as Xuan Shen (*Scrophularia ningpoensis*), Mai Dong, and Sheng Di Huang [16].

However, the underlying molecular mechanisms which drive the SS pathogenesis have not been fully elucidated, and genetic factors, viral infections, immune system exceptions, inflammatory infiltration, neural hormones, and environmental factors are considered to be the important factors that cause the disease [32]. Due to the complexity of this disease, its treatment has always been a difficult point in research. Not a recognized specific drug has been available in treating SS in the market, and most of them adopt symptomatic treatment, but all of them have problems such as uncertain efficacy and large toxic side effects [12]. Therefore, a novel and potent therapy for SS treatment is in urgent need from a broader perspective. The extensive application of “compound-proteins” pathways have become the TCM network pharmacology research hotspot, allowing to vividly interpreting the complicated interactions of biological systems, drugs, and diseases [33]. Because of these characteristics, network pharmacology has been considered the next paradigm in drug discovery. In this study, network pharmacology was used to analyze the active components of QZF in the treatment of SS and to predict its therapeutic target and pathway, so as to provide theoretical support for its clinical effectiveness.

In the early stages of SS, CD4+ T cells, B cells, CD8+ T cells, macrophages, and dendritic cells are the infiltrating cells in lacrimal and salivary glands, and among these cells, CD4+ T cells are the most common [34]. Th17 cells, a subset of CD4+ memory effector T cells, secrete IL-17A and IL-17F under the stimulation of TGF-*β* and IL-6 stimulation [35]. It was noted that increased IL-17 levels were found in minor salivary glands of SS patients, and the expression of IL-17 is correlated with the severity of inflammation [36]. No disease development was observed in IL-17 knockout mice, and mice models of SS showed the strongest evidence of IL-17 and Th17 in SS disease [37]. IL-6, IFN-*γ*, and IL-1 levels are also increased in serum, saliva, and tears of SS patients [38]. IL-10 has a rather anti-inflammatory or protective action toward glandular injury in SS [11]. Furthermore, a statistically significant overall decrease in mean IL-10 levels occurs, while IL-6 and TNF-*α* levelssignificantly increased in pSS patients [39]. Results of in vivo showed that IL-6, IL-17A, and TNF-*α* were upregulated in serum and submandibular gland of SS mice, and IL-10 was downregulated, which was consistent with previous research. In SS mice treated with QZF, levels of IL-6, IL-17A, and TNF-*α* were reduced while IL-10 levels increased. GO analysis of the QZF-therapeutic potential target genes also showed that CD4-positive T cell cytokine production, Th17 cell differentiation, and Th17 type immune response were important GO terms. TNF, IL-6, IL-1B, and IL-10 are the top four targets in KEGG analysis. However, our results showed no significant difference in IFN-*γ* and IL-1 in QZF-treated SS mice. These results suggest that QZF maybe regulate the expression of TNF-*α*, IL-17A, IL-6, and IL-10 to treat SS.

KEGG analysis showed that the related pathways of QZF in the treatment of SS included the FOXO signaling pathway, *Yersinia* infection, TNF signaling pathway, and HIF-1 signaling pathway. There are 20 pathways, which are closely related to inflammatory response, oxidative stress, autophagy, apoptosis, and submandibular gland cell signaling. A large number of experimental studies have shown that the FOXO family of transcription factors plays a crucial role in many basic cellular processes such as immune regulation, metabolism, proliferation, apoptosis, and oxidative stress response [40–42]. Inhibition of FOXO1 can reduce the expression of TNF-*α* and IL-6 [43]. Moreover, pSS patients had significantly higher levels of the proinflammatory cytokines IL-17, IL-6, and TNF-*α* along with a significant decrease in the anti-inflammatory cytokine IL-10 and FOXP3 mRNA expression compared with healthy controls [39]. Many studies have shown that hypoxia-inducible factor-1 (HIF-1) pathway is involved in the pathogenesis of autoimmune diseases, such as rheumatoid arthritis, systemic lupus erythematosus, and SS [44–47]. Inhibition of HIF-1*α* can prevent local capillary hyperplasia and reduce CD4+T differentiation to Th17 cells, thereby reducing inflammatory response and reducing apoptosis of exocrine glands such as lacrimal salivary glands and salivary glands [45]. Akt is one of the important components of PI3K/Akt pathway, which is closely related to cell survival, apoptosis, and other biological activities [48]. Phosphorylated Akt can enhance the activity of HIF-1*α*, thereby initiating the transcription of HIF-1*α*-related target genes and ultimately enhancing cell proliferation and inhibiting cell apoptosis [49]. These findings suggest that HIF-1 is closely related to the PI-3K/Akt signaling pathway, which has potential research value in the treatment of immune diseases. In addition, it has been reported that HIF-1 can affect the ratio of Th17 and Treg cells and then cause the immune imbalance of Th17 and Treg cells [50]. In this study, levels of Akt and HIF-1*α* and apoptotic rate were significantly decreased in QZF-treated SS mice. It is implied that QZF may regulate apoptosis of the submandibular gland tissue through these pathways, thus slowing down SS.

## 5. Conclusion

In this work, we proposed a network pharmacology approach, combining SS mice model to explore the efficiency of QZF for the treatment of SS. To sum up, our results suggested that the QZF might mainly regulated inflammatory response, chemokine metabolic process, immune system, and cell apoptosis by the FOXO signaling pathway, TNF signaling pathway, and HIF-1 signaling pathway to play an important role in the treatment of SS. Akt1, HIF-1*α*, TNF-*α*, IL-17A, IL-6, and IL-10 may be the key target gene of QZF in the treatment of SS. Stigmasterol, neocryptotanshinone II, neotanshinone C, miltionone I, and beta-pinene may be the important active ingredients of QZF in the treatment of SS. These results demonstrated that QZF had the characteristics of multicomponent, multitarget, and multipathway therapy for SS, providing potential drugs and targets for the treatment of SS.

## Data Availability

The data supporting the findings of this study are available from the corresponding author upon request.
